# Beyond the Ohmic Limit: Co-Ion Control and Conductance Scaling Regimes in Rectifying Nanochannels

**DOI:** 10.1021/acs.jpclett.6c02625

**Published:** 2026-09-25

**Authors:** María Queralt-Martín, Elena García-Giménez, Jose Joaquín Pérez-Grau, Antonio Alcaraz

**Affiliations:** † Laboratory of Molecular Biophysics. Department of Physics, University Jaume I, 12071 Castellón, Spain; ‡ Instituto de Ciencia Molecular, Universitat de València, Calle Catedrático José Beltrán Martínez 2, 46980 Paterna, Spain

## Abstract

The power-law scaling of ionic conductance *G* ∼ *c* ^α^ serves as a fundamental probe of transport physics in confined geometries, where transport is traditionally restricted to sublinear regimes (α < 1) governed by majority counterions. While current rectification is widely exploited in nanofluidic diodes and iontronic circuits, its direct consequence on this scaling law remains unexplored. Here, we show that moving beyond the ohmic limit establishes a co-ion control paradigm that dictates transport in asymmetric nanoenvironments. By pairing an analytical electrodiffusion framework with experimental validation across a diverse spectrum of heterogeneous systems, including bipolar biological channels (OmpF, gramicidin A) and unipolar synthetic conical nanopores, we demonstrate a polarity-driven transition in ionic control. In the rectifying polarity, the formation of an electrostatic depletion zone transfers transport control from majority counterions to minority co-ions. Remarkably, this electrostatic reconfiguration yields supralinear scaling (α > 1) in bipolar configurations while driving unipolar ones toward linearity (α ∼ 1). By unifying biological and solid-state membranes under a single predictive model, these findings clarify how pore geometry, charge asymmetry, and voltage polarity cooperatively govern nanoscale ion transport.

## Graphical abstract

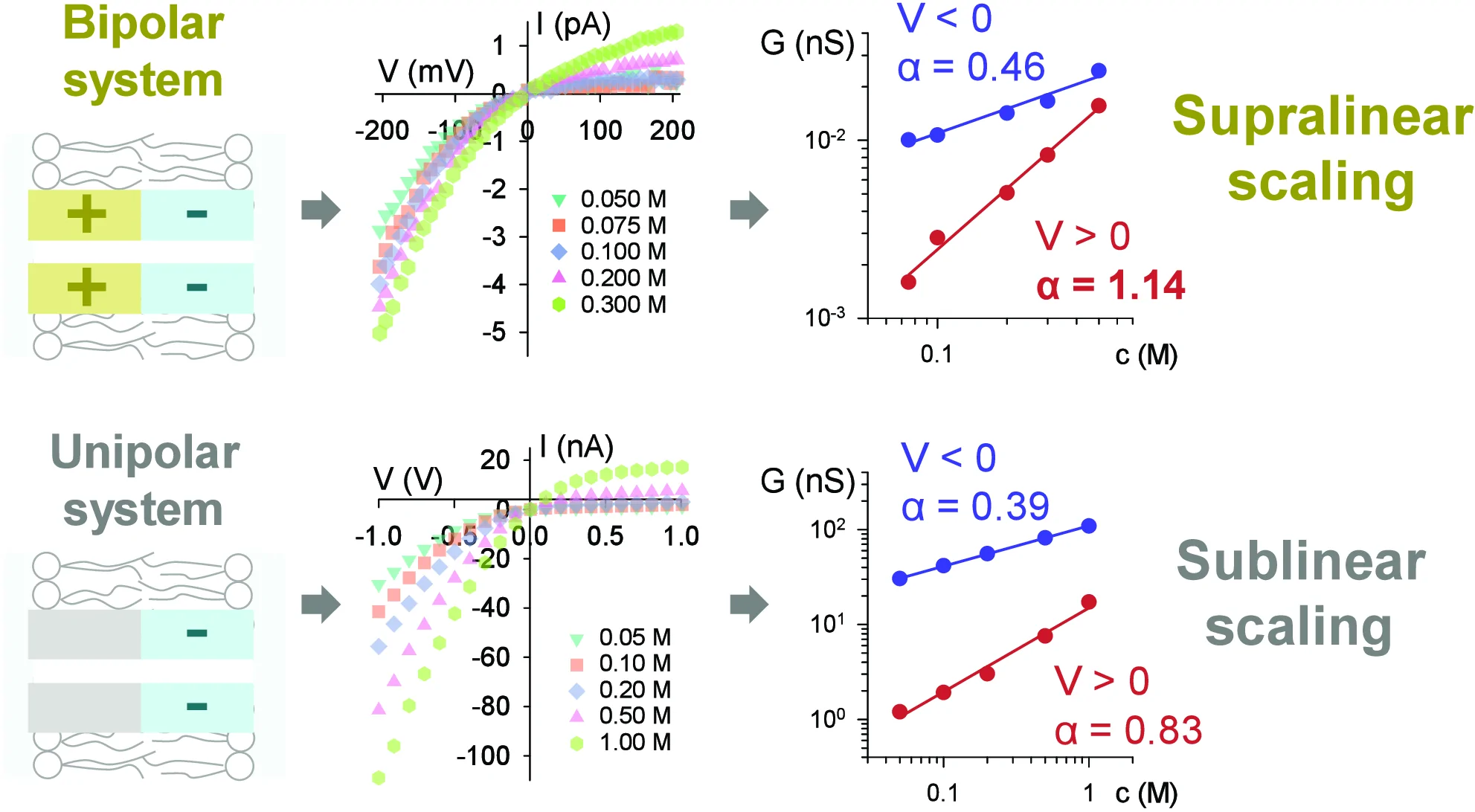

As a fundamental diagnostic of nanoscale ionic transport, the relationship between pore conductance (*G*) and concentration (*c*) typically follows a power-law relationship *G* ∼ *c* ^α^.^1–4^ Extensive research in ohmic pores shows that the scaling exponent α is far more than a mere curve-fitting parameter; it directly reflects the underlying physicochemical landscape, capturing both hydrodynamic constraints (via access resistance)^5,6^ and the electrostatic interactions between pore surface charges and permeating ions.^1,4^ While scaling exponents are traditionally constricted between 0 and 1,^2,4^ recent evidence shows that co-ion transport can drive supralinear scaling in particular arrangements that, despite including separate clusters of opposite charge,^7^ still maintain ohmic conduction. Here, we move beyond the ohmic limit to establish the co-ion control paradigm as a universal mechanism governing transport in asymmetric nanoenvironments. To demonstrate the generality of this framework, we examine current–voltage *I*–*V* characteristics across a spectrum of structurally and chemically inhomogeneous pores—ranging from bipolar charge distributions in biological channels (OmpF, gramicidin A) to unipolar synthetic conical nanopores—uncovering a polarity-driven handover in ionic governance.

First, we consider the bacterial porin OmpF from *E. coli*, a protein that forms wide, characteristically ohmic channels under symmetrical pH conditions.^8^ However, imposing a trans-membrane pH gradient subjects the pore residues to an asymmetric titration, establishing a bipolar fixed charge distribution that triggers pronounced current rectification.^9–11^ Utilizing an experimental configuration with pH 3 in the cis compartment and pH 12 in the trans compartment (Figure 1A, left panel) yields the rectifying *I*–*V* curves illustrated in Figure 1A (right panel). The resulting channel conductance (*G* = *I*/*V*), evaluated at a representative rectifying bias of ±130 mV across a range of salt concentrations, reveals a distinctive concentration dependence that is shown in the lower panel of Figure 1A.

**Figure 1:**
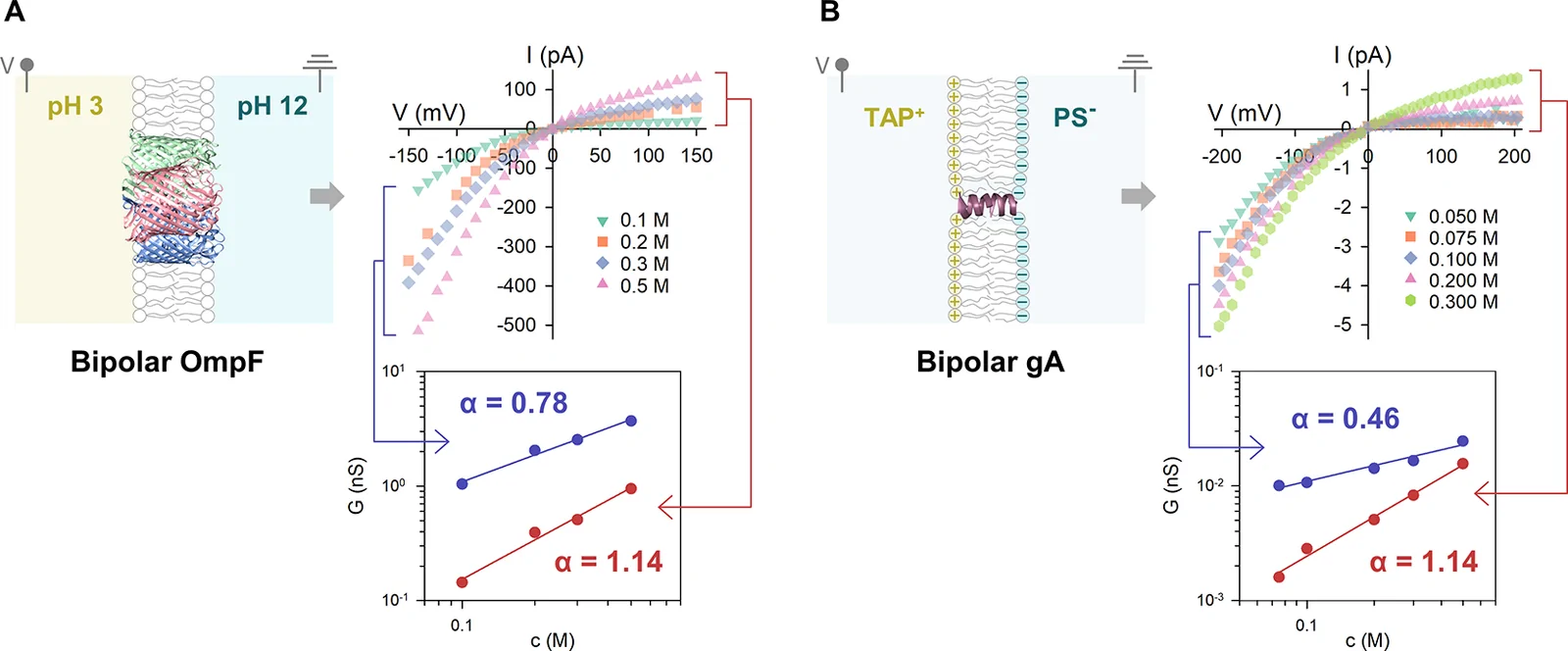
Conductance scaling with concentration in bipolar systems. (A) Cartoon depicting OmpF inserted under a bipolar configuration generated with a salt solution at pH 3 at one side of the membrane and pH 12 at the other side (left panel); *I*–*V* curves measured at different salt concentrations for the configuration displayed (right panel), and resulting conductance scaling with concentration at |*V*| = 130 mV (lower panel). (B) Cartoon illustrating *gA* inserted under a bipolar configuration obtained by using an asymmetric lipid membrane with positively charged DOTAP (TAP^+^) at one side of the membrane and negatively charged DPhPS (PS^–^) at the other side (left panel); *I*–*V* curves measured at different salt concentrations for the configuration displayed (right panel), and resulting conductance scaling with concentration at |*V*| = 100 mV (lower panel). In the *G* vs *c* plots, solid lines indicate power fits *G* ∝ *c* ^α^.

Under the conductive polarity (*V* < 0 in the *I*–*V* curves of Figure 1A), the scaling exponent for conductance is α ≈ 0.8 (Figure 1A, lower panel), consistent with the sublinear scaling typically observed in ohmic pores and classically attributed to counterion accumulation driven by protein and membrane surface charges.^2^ Conversely, under the rectifying polarity, α increases systematically with the applied voltage, exceeding unity to reach supralinear scaling regimes (α ≈ 1.14) at high biases. This behavior is consistent with the supralinear scaling previously reported in particular ohmic systems that also feature bipolar configurations, such as cation-selective channels embedded in positively charged lipid bilayers, or anion-selective channels in negatively charged ones.^7^

To demonstrate that this scaling behavior is independent of protein titration or pH fluctuations, we next examine a rectification mechanism arising purely from asymmetric membrane electrostatics. This is achieved by reconstituting the channel-forming peptide gramicidin A (gA) from *B. brevis* into asymmetric lipid bilayers composed of positively charged DOTAP in one leaflet and negatively charged DPhPS in the other (Figure 1B, left panel).^12^ Although gA typically forms ohmic pores, this interfacial membrane asymmetry induces a highly rectifying *I*–*V* curve (Figure 1B, right panel). The conductance scaling for gA closely mirrors the trends observed for OmpF (Figure 1B, lower panel). Specifically, the scaling exponent in the conductive branch (*V* < 0) is α ≈ 0.5, whereas under the rectifying polarity, it climbs to α ≈ 1.14.

To interpret these experimental findings, we employ a theoretical framework rooted in the Poisson–Nernst–Planck (PNP) formalism, as formulated for structureless membranes^13^ and biological ion channels.^2,7,14^ This coupled set of algebraic and differential expressions constitutes a nonlinear boundary value problem that typically requires numerical resolution via iterative computational schemes once the channel’s internal electrostatic landscape and fixed charge distribution are known.^15,16^ However, the experiments in Figure 1 demonstrate that pH gradients and charged lipid membranes alter the ionization state of the channel charged residues, a phenomenon current methodologies can only partially capture by assuming hypothetical charge distributions and arbitrary parameter fits.^15,17^ To address this, we derive an analytical solution that clarifies transport dynamics in key limiting cases, albeit at the cost of making significant simplifications. Specifically, we adopt the Goldman constant-field approximation across a homogeneous membrane of thickness *d*. Under these steady-state assumptions, the ionic flux density for a given permeating species *i* = +,– is analytically resolved as

$$J_{i}\left(V\right)=-\left(\frac{z_{i}FD_{i}\varphi_{\mathrm{dif}}}{RTd}\right)\frac{c_{i}{\left(0\right)e^{{\;\;z}_{i}F\varphi_{\mathrm{dif}\;}/RT}-c}_{i}\left(d\right)}{1-{\;e}^{z_{i}F\varphi_{\mathrm{dif}}/RT}}$$ 1

where *D*_*i*_ is the diffusion coefficient of species *i* in the pore solution, and constants *F*, *R*, and *T* have their usual meaning,^18^ φ_dif_ is the diffusion potential, and

2 $${c_{i}\left(o\right)=c}_{\mathrm{cis}}\;e^{{-z}_{i}F\varphi_{\mathrm{cis}}/RT}$$

3 $${c_{i}\left(d\right)=c}_{\mathrm{trans}}\;e^{{-z}_{i}F\varphi_{\mathrm{trans}}/RT}$$

correspond to Donnan equilibrium conditions at the membrane/solution interfaces, being *c*_cis_ and *c*_trans_ the bulk concentrations in these compartments.^7,14^ Considering that *V* = φ_cis_ + φ_dif_ – φ_trans_ is the total applied voltage through the system, the total current is given by

4 $$I=FA_{\mathrm{eff}}\left(J_{+}\left(V\right)+J_{-}\left(V\right)\right)$$

where *A*_eff_ is the effective pore cross-sectional area.^12^ This analytical formulation yields rectifying *I*–*V* curves using φ_cis_ and φ_trans_ as effective parameters that encapsulate not only the electrostatic influence of both protein and membrane charges,^12,13,19^ but could also account for interfacial effects such as access resistance.^2,5^ Extensive literature shows that access resistance dominates channel conductance when the channel aspect ratio (length-to-diameter ratio) is close to one, as is the case for OmpF, but only in dilute solutions.^5^ In long channels such as micrometric-long synthetic nanopores, or very narrow channels such as gramicidin A, access resistance is irrelevant.^2^ Thus, in our calculations involving moderately concentrated solutions, access resistance is omitted, and the specific charge distribution is implemented using fixed charges *X*_cis_ = 500 mM and *X*_trans_ = −500 mM, yielding the resulting axial electrical potential profile illustrated in Figure 2A. The corresponding *I*–*V* curves generated by this framework are displayed in Figure 2B (upper panel).

**Figure 2:**
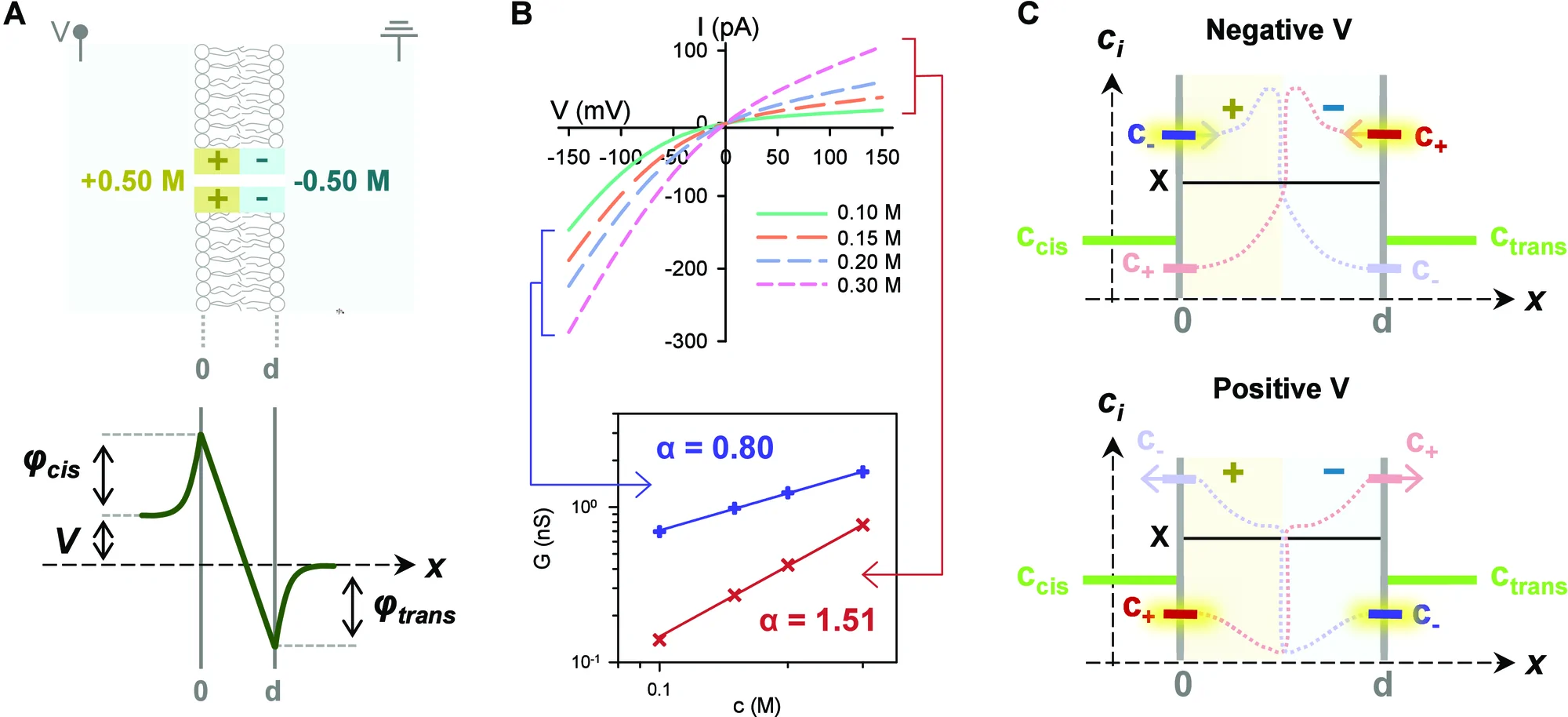
Theoretical model with analytical solutions to describe current rectification and conductance scaling with concentration in bipolar systems. (A) Cartoon depicting the bipolar system (upper panel) and schematic illustration of the corresponding potential distribution along the channel axis (lower panel). (B) *I*–*V* curves obtained with the model in the configuration depicted in panel (A) and resultant scaling of conductance with concentration at each polarity. The solid lines indicate power fits *G* ∝ *c* ^α^. (C) Schematic representation of the ion concentration profiles that arise from the bipolar configuration used and the voltage applied. Under negative polarities (upper panel), abundant counterions at each side (*c*_–_(0) and *c*_+_(*d*)) are impelled into the channel and become accumulated at the interface to avoid accessing the region with opposite fixed charge. Co-ions (*c*_+_(0) and *c*_–_(*d*)) see their concentrations at the interface also increased to fulfill electroneutrality. Conversely, when a positive voltage is applied (lower panel), counterions at each side move away from the channel, causing ion depletion at the interface, and so do co-ions to fulfill electroneutrality. Highlighted in yellow are the ions that dominate the conductance scaling at each voltage, as derived from eqs 5 and 6 (counterions at negative voltage and co-ions at positive polarity).

The calculated conductance-concentration *G*–*c* relationships for the fixed-charge distributions used are shown in Figure 2B (lower panel). In agreement with the experimental findings, under positive voltages, the scaling exponent is supralinear, whereas it remains sublinear in the conducting branch under negative biases. To elucidate the physical origin of these distinct scaling regimes, we derive the asymptotic limits of the channel conductance (*G*_lim_) by combining eqs 1–4. Under a sufficiently high negative applied bias, this asymptotic scaling reduces to

5 $$G_{\lim}^{-}=\frac{I\left(-V\right)}{-V}\approx \pi r^{2}\frac{FD_{+}}{RTd}c_{+}\left(d\right)+\pi r^{2}\frac{FD_{-}}{RTd}c_{-}\left(0\right)\approx \pi r^{2}\frac{FD_{+}}{RTd}c_{\mathrm{trans}}\;e^{-F\varphi_{\mathrm{trans}}/RT}+\pi r^{2}\frac{FD_{-}}{RTd}c_{\mathrm{cis}}\;e^{F\varphi_{\mathrm{cis}}/RT}$$

Given that we have set φ_cis_ > 0 and φ_trans_ < 0, the conductance in the conducting polarity *G*_lim_^–^ is fundamentally ruled by counterion concentrations at both membrane boundaries. This distribution is illustrated schematically in Figure 2C (upper panel, yellow-highlighted *c*_–_ on the left side and *c*_+_ on the right side, where the system has a positive and negative fixed charge, respectively).^10,20^ Under these conditions, the two dominant contributions to *G*_lim_^–^ follow the classical Donnan-like regime where the local counterion concentration scales nonlinearly with bulk concentration. Consequently, the resulting scaling exponent transitions smoothly between α = 0 in dilute solutions and α = 1 in concentrated ones.^2^ In contrast, for high enough positive applied voltages,

6 $$G_{\lim}^{+}=\frac{I\left(V\right)}{V}\approx \pi r^{2}\frac{FD_{+}}{RTd}c_{+}\left(0\right)+\pi r^{2}\frac{FD_{-}}{RTd}c_{-}\left(d\right)\approx \pi r^{2}\frac{FD_{+}}{RTd}c_{\mathrm{cis}}\;e^{-F\varphi_{\mathrm{cis}}/RT}+\pi r^{2}\frac{FD_{-}}{RTd}c_{\mathrm{trans}}\;e^{F\varphi_{\mathrm{trans}}/RT}$$

the conductance in the rectifying polarity *G*_lim_^+^ becomes dictated by co-ion concentrations at both membrane boundaries (highlighted in yellow in Figure 2C, lower panel). Note that co-ion concentration scales quadratically with bulk concentrations in the dilute solution limit.^7^ As a consequence, this electrostatically depleted regime breaks the traditional sublinear constraint, driving the supralinear scaling response (α ≈ 1.14) observed experimentally:

7 $$c_{\mathrm{cis}}\;e^{-F\varphi_{\mathrm{cis}}/RT}=\left(-\frac{X_{\mathrm{cis}}}{2}+\sqrt{{\left(\frac{X_{\mathrm{cis}}}{2}\right)}^{2}+{c_{\mathrm{cis}}}^{2}}\right)\approx \frac{{c_{\mathrm{cis}}}^{2}}{X_{\mathrm{cis}}}$$

8 $$c_{\mathrm{trans}}\;e^{F\varphi_{\mathrm{trans}}/RT}=\left(-\frac{X_{\mathrm{trans}}}{2}+\sqrt{{\left(\frac{X_{\mathrm{trans}}}{2}\right)}^{2}+{c_{\mathrm{trans}}}^{2}}\right)\approx \frac{{c_{\mathrm{trans}}}^{2}}{X_{\mathrm{trans}}}$$

In the general case, the effective scaling exponent obtained from eq 6 decreases progressively from α ≈ 2 as the electrolyte concentration increases, asymptotically approaching α ≈ 1 while strictly maintaining values of α > 1.

Beyond biological channels, the physical origin of current rectification in bipolar membranes has fueled intense debate for decades.^21^ Classical analytical expressions for the limiting current predict a quadratic dependence on salt concentration, operating on the assumption that the junction interface undergoes complete co-ion depletion.^20,22–25^ Experimentally, however, this ideal quadratic scaling is rarely observed. Even in state-of-the-art bipolar membranes engineered with high fixed-charge densities for electromembrane processes, the scaling exponent for the rectified current typically spans a range between 1 and 1.5 ^20,23^ (Figure S1), closely matching the supralinear regimes observed in our experiments (Figure 1). Traditionally, this discrepancy has been attributed to deviations from the abrupt-junction model, which assumes steplike charge distributions.^26^ In practice, realistic bipolar fluidic systems usually involve gradual junction profiles or neutral transition zones. These features decrease the maximum electric field and broaden the depletion region at the interface, leading to incomplete co-ion exclusion.^21,26–29^ Ultimately, the underlying debate remains deeply intertwined with the fundamental physics of co-ion self-diffusion in charged polymeric membranes.

As exhaustively reviewed in the literature,^18,30^ abundant experimental evidence across a variety of membrane systems reveals substantial deviations from standard Donnan theory. Early attempts to reconcile these discrepancies involved introducing activity coefficients into the theoretical framework, or accounting for experimental solvent transfer, electro-osmosis, and enhanced ion pairing within the membrane phase.^30^ Glueckauf and co-workers investigated the issue in detail, even utilizing radioactive tracers to measure electrolyte uptake in ion-exchange membranes.^31^ For systems with 1:1 electrolytes, they observed a scaling exponent of approximately 1.3–1.4, a value that deviates significantly from the 2.0 predicted by standard, ideal Donnan theory (eqs 7 and 8). Because the Donnan equilibrium law strictly applies to a pair of homogeneous phases, they concluded that the membrane phase must be inherently heterogeneous.^31^ This reasoning aligns with a co-ion control mechanism operating in biological nanochannels, which typically feature constrained, irregular geometries and corrugated amphoteric surfaces.^32^ Consequently, this structural heterogeneity provides a plausible physical origin for the supralinear scaling exponents observed in the positive branches of Figure 1.

While we acknowledge that the simplifications of our analytical treatment are substantial, considering only two homogeneously charged regions of opposite polarity, a linear potential profile between them, and local Donnan equilibrium, the universality and predictive power of this approach can be rigorously tested when applied to unipolar rectifying systems, such as synthetic conical nanopores. Figure 3A displays the *I*–*V* curves (right panel) measured using a track-etched polyimide conical nanopore^33^ (see left panel for a schematic representation) alongside the resulting scaling behavior *G*–*c* (lower panel). Remarkably, the scaling exponent obtained in the conductive branch (*V* < 0) is around α ∼ 0.4, whereas under the rectifying polarity, the scaling remains sublinear at α ≈ 0.8, clearly below unity, in stark contrast to the supralinear exponents observed in the bipolar configurations of Figure 1.

**Figure 3:**
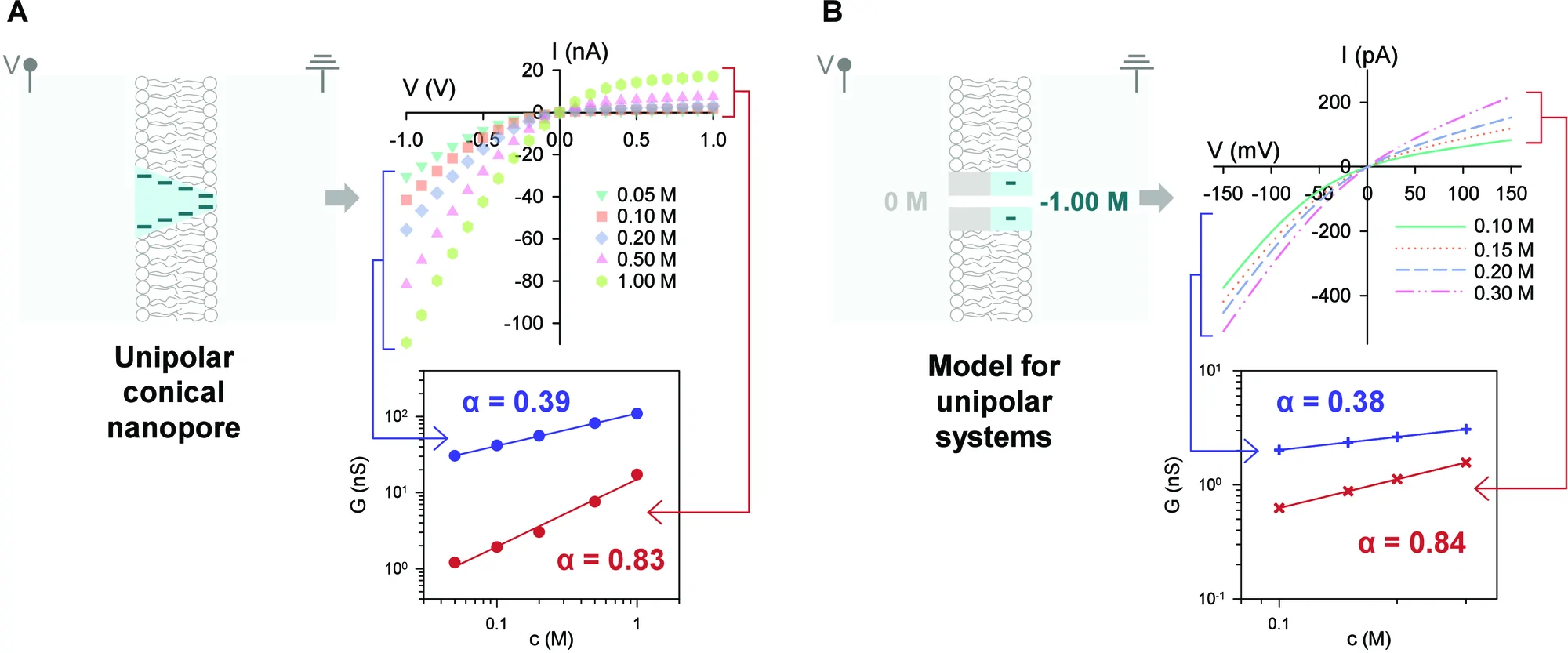
Conductance scaling with concentration in a unipolar system. (A) Cartoon depicting the unipolar system (left panel), *I*–*V* curves measured at different salt concentrations (right panel) and resulting conductance scaling with concentration (lower panel) for a polyimide conical synthetic nanopore. (B) Theoretical model with analytical solutions to describe current rectification and conductance scaling with concentration in unipolar systems. The cartoon depicting the unipolar system is shown on the left, while the *I*–*V* curves and conductance scaling with concentration derived from the model are shown on the right. In the *G* vs *c* plots of panels (A) and (B), solid lines indicate power fits *G* ∝ *c* ^α^.

Within our theoretical framework, a conical nanopore can be modeled using two effective charges of the same sign: a high fixed-charge density at the narrow tip aperture and a lower one at the wide base.^34^ Here, to achieve current rectification under positive polarity, the region with the higher fixed-charge density is positioned adjacent to the trans compartment. A representative calculation of this rectifying behavior, simulated with *X*_cis_ = 0 and *X*_trans_ = −1000 mM, is presented in Figure 3B. Of note, the theoretical scaling profiles generated by this model are in excellent agreement with the experimental observations in Figure 3A, yielding a strictly sublinear scaling exponent in the rectifying polarity. The physical basis for this response is readily elucidated by eqs 5 and 6. Because *X*_cis_ and *X*_trans_ share the same polarity now, the conductance in the conducting state (*V* < 0) remains governed by counterion accumulation within the narrow tip region on the trans side. In contrast, the conductance under the rectifying polarity (*V* > 0) is dictated by co-ion concentration in the trans side but governed by the counterion concentration on the cis side. Given that *X*_cis_ ≈ 0, counterions and co-ions are similarly abundant at the cis boundary, causing the overall scaling response to approach that of the bulk electrolyte.

In summary, we combined experimental measurements across diverse nanofluidic systems with a 1D analytical electrodiffusion framework to demonstrate how fixed-charge distributions govern the scaling behavior of rectified ionic currents. Our analysis of the power-law scaling *G* ∼ *c* ^α^ under opposing voltage polarities shows that the scaling exponent α is a direct reflection of the ionic species controlling the transport. Specifically, our study transitions from biological channels featuring bipolar charge distributions—implemented via a trans-membrane pH gradient in OmpF or through asymmetric lipid leaflets in gramicidin A—to synthetic unipolar architectures, represented by track-etched polyimide conical nanopores. While the bipolar systems reveal a polarity-driven handover that triggers supralinear scaling (α > 1) due to a co-ion bottleneck in the rectifying state, the unipolar conical pores maintain sublinear regimes (α < 1) as transport control shifts toward bulk-like boundaries. By unifying these distinct physical responses within a single analytical model, this work provides a framework that bridges biological and solid-state membranes, clarifying the cooperative role of pore geometry, charge asymmetry, and voltage polarity in nanoscale ion transport.

## Experimental Methods

OmpF and gA experiments were carried out as described elsewhere^2,12,33^ by forming a planar lipid bilayer that separates two compartments filled with electrolyte solutions and inserting one or several channels. For experiments with OmpF, membranes were formed from diphytanoylphosphatidyl choline (DPhPC). For gA, lipid bilayers were built by using dioleoyl trimethylammonium propane (DOTAP) on the cis side and diphytanoylphosphatidyl serine (DPhPS) on the trans side. All lipids were from Avanti, Inc. (Alabaster, AL, USA). OmpF was a kind gift of Dr. S. M. Bezrukov (NIH, Bethesda, MD, USA). gA was obtained by purchasing a natural mixture of gramicidins and separating gA by high-performance liquid chromatography (HPLC). Experiments with the polyimide conical nanopore were performed as outlined in ref 33. The nanopore was fabricated by foil irradiation with heavy ions and connected two compartments that were filled with symmetrical KCl solutions at different concentrations and pH 6.5–7.0. More information about the experimental methods is provided in the Supporting Information.

## Supplementary Material



## Notes

Supplementary figure showing conductance scaling with concentration from two different synthetic bipolar membranes; supplementary methods provide additional details about the experimental procedure (PDF)
