# Supplementary material for: Beyond the Ohmic Limit: Co-Ion Control and Conductance Scaling Regimes in Rectifying Nanochannels
Source: J Phys Chem Lett. 2026 Sep 25;17(40):11391–6. doi: 10.1021/acs.jpclett.6c02625 (PMC13656771; doi:10.1021/acs.jpclett.6c02625)
Supplement: Supplementary file 1 [file jz6c02625_si_001.pdf]

Supporting Information

Beyond the Ohmic Limit: Co-Ion Control and Conductance Scaling Regimes in Rectifying Nanochannels

María Queralt-Martín[1], Elena García-Giménez[1], Jose Joaquín Pérez-Grau[2], Antonio Alcaraz[1]*

[1]Laboratory of Molecular Biophysics. Department of Physics. University Jaume I, 12071 Castellón, Spain

[2]Instituto de Ciencia Molecular, Universitat de València, Calle Catedrático José Beltrán Martínez 2, 46980, Paterna, Spain

*Corresponding author: alcaraza@uji.es

Supplementary Figure

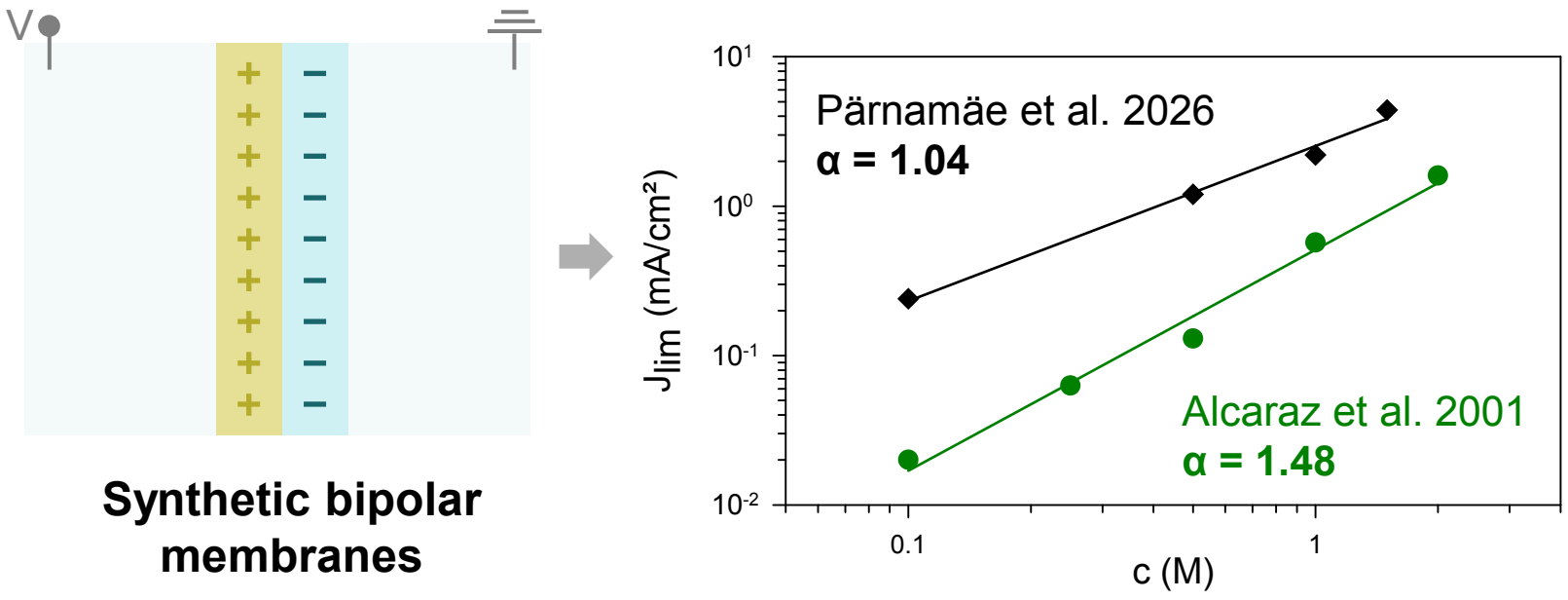

Figure S1. Conductance scaling with concentration from two different synthetic bipolar membranes, obtained with permission from Pärnamäe et al. 2026 and from Alcaraz et al. 2001.

Detailed experimental methods

Planar lipid membranes for OmpF and gA experiments were formed by using the solvent free modified Montal-Müller technique [34,35]. Briefly, planar membranes were formed by the apposition of lipid monolayers across an orifice with a diameter of ~100 µm on a 15 mm-thick Teflon film. The film separated two 1.8-mL compartments (so-called *cis* and *trans*) of a Teflon chamber. Lipids were prepared by dissolving them in pentane (Baker) at 5 mg/mL after chloroform evaporation. Aliquots of 10–20 µL of lipid in chloroform were added on top of the salt solution in each compartment. The orifice was pre-treated with a 3% solution of hexadecane in pentane. After pentane evaporation, the level of solutions in each compartment was raised above the hole so the planar bilayer could form by apposition of the two monolayers.

For OmpF, channel reconstitution was achieved by adding 0.1 µL of OmpF protein at 1 ng/mL in 1 M KCl and 1% (v/v) OctylPOE (Alexis, Switzerland) to the *cis* side of the membrane. Solutions consisted of KCl at different concentrations with the pH set at 3 (*cis*) or 12 (*trans*) by adding HCl or KOH. pH was monitored during the experiments

using a GLP22 pHmeter (Crison). OmpF was a kind gift of Dr. S. M. Bezrukov (NIH, Bethesda, MD, USA).

For gA experiments, gramicidin from *Bacillus brevis* was purchased as a mixture of the naturally occurring gramicidins A, B, C, and D from Sigma-Aldrich (code G5002). To further purify gA out of the other gramicidins in the mixture, 7 μL of methanolic sample (1 mg/mL) were injected into a 4.6 x 250-mm "Zorbax-C8" column. The column was eluted at 1.5 mL/min (pressure -1,800 psi) using 80% methanol that had been filtered and degassed with helium. The gramicidins in the eluant were detected by UV absorbance at 280 nm. When the polarity of the carrier solvent is increased, the gramicidin A peaks are completely separated. The valine gramicidin A peak was collected by hand, concentrated by the evaporation of solvent, and purified a second time on the same HPLC column using 81% methanol. The final sample was stored in glass with room air at -20°C. For electrophysiology experiments, gA was diluted in ethanol at 10^{-7} M and 1-5 μL were added into both the *cis* and *trans* compartments. Then membrane was reformed to favor gA partition into the bilayer. Solutions were symmetrical and consisted of KCl at pH 6 with 1 mM EDTA.

The polyimide nanopore (PI NP) used in our experiments was fabricated by foil irradiation with heavy ions as explained previously [36,37]. Subsequently, the ion tracks were converted into nanopores by exposing the polymer membrane to chemical etching with a strong inorganic etchant under asymmetric conditions. The membrane was placed in an electrochemical cell separating two salt solutions. The solution pH was in the range 6.5−7.0 during measurements. Because these pH values are higher than the pKa characteristic of the −COOH moieties functionalized on the pore surface, the carboxylic acid groups were ionized to $-COO^-$ and thus the pore was negatively charged. The resulting nanopore has an approximate conical shape (so called bullet-like). The radius of the pore base was obtained by direct imaging of the region, while pore tip radius was estimated from the nanopore electrical conductance, obtaining ~100 nm (base) and ~15 nm (tip). The nanopore was placed in the electrophysiology setup so that its tip (narrow opening) faced the *cis* side.

The chamber for OmpF and gA or that with the nanopore was connected to an Axopatch 200B amplifier (Molecular Devices, Sunnyvale, CA) in the voltage-clamp mode through Ag/AgCl electrodes covered with 2 M KCl, 1.5% agarose bridges assembled within standard 250 μL pipette tips. The working electrode was placed in the *cis* side, whereas the *trans* side was set to ground. Thus, the potential is defined as positive when it is higher on the *cis* side. Data from the amplifier were filtered by an integrated low pass Bessel filter at 10 kHz (1 kHz for gA experiments), digitized with a Digidata 1440A (Molecular Devices, Sunnyvale, CA) at a sampling frequency of 50 kHz and analyzed using pClamp 10.7 software (Molecular Devices, Sunnyvale, CA). The chamber and the head stage were isolated from external noise sources with a double metal screen (Amuneal Manufacturing Corp., Philadelphia, PA). All measurements were performed at room temperature. Current values for I-V curves were obtained from current traces using the Gaussian fit tool of Clampfit 10.7 (Molecular Devices, Sunnyvale, CA).
